# Neuroimaging in Cerebral Palsy – Report from North India

**Published:** 2013

**Authors:** Anju AGGARWAL, Hema MITTAL, Sanjib KR DEBNATH, Anuradha RAI

**Affiliations:** 1 Department of Pediatrics, University College of Medical Sciences, Delhi, India; 2 Guru Tegh Bahadur Hospital, Delhi, India

**Keywords:** Cerebral Palsy, Neuroimaging, Birth asphyxia, Children

## Abstract

**Objective:**

Only few Indian reports exist on neuroimaging abnormalities in children with cerebral palsy (CP) from India.

**Materials & Methods:**

We studied the clinico-radiological profile of 98 children diagnosed as CP at a tertiary centre in North India. Relevant investigations were carried out to determine the etiology.

**Results:**

Among the 98 children studied, 80.5% were males and 22.2% were premature. History of birth asphyxia was present in 41.9%. Quadriplegic CP was seen in 77.5%, hemiplegic in 11.5%, and diplegic in 10.5%. Other abnormalities were microcephaly (60.5%), epilepsy (42%), visual abnormality (37%), and hearing abnormality (20%). Neuroimaging was abnormal in 94/98 (95.91%).

Abnormalities were periventricular white matter abnormalities (34%), deep grey matter abnormalities (47.8%), malformations (11.7%), and miscellaneous lesions (6.4%). Neuroimaging findings did not relate to the presence of birth asphyxia, sex, epilepsy, gestation, type of CP, or microcephaly.

**Conclusions:**

Neuroimaging is helpful for etiological diagnosis, especially malformations.

## Introduction

Cerebral palsy (CP) is a common cause of severe motor disability in India. Data on clinical, etiological, and neuroimaging profile from India is sparse (1,2). Studies reporting the neuroimaging findings in children with CP in India are few compared to the West (3,4). Neuroimaging may suggest underlying etiology, extent and time of brain damage, which are helpful for diagnostic, rehabilitative, and preventive services in these children. The aim was to study the clinical and radiological profile of children diagnosed with CP at a tertiary centre in north India.

## Materials & Methods

The study was conducted at the Neurodevelopmental Clinic of the Department of Pediatrics at a tertiary care hospital in north India over two-year period 2008-2010 and approval was taken from the hospital ethical committee. A written informed consent was taken from the parents of the subjects. The Study included 98 children diagnosed as CP who underwent 3 Tesla MRI. T1, T2 and flair sequences were performed, and both coronal and axial sections were studied. All children were diagnosed and managed as per a standard protocol. Baseline demographic data and detailed history were recorded, andphysical and neurological examination was carried out. CP was diagnosed according to a standard clinical definition (5). As per WHO database definition for Southeast Asia region, birth asphyxia was defined as slow gasping breathing or no breathing at 1 minute of age (for extramural babies) and Apgar less than 7 at one minute (for intramural babies) (6). Neuroimaging findings were classified similar to a previous study by Krägeloh-Mann et al. (4). The categories were classified into three groups based on etiopathogenetic patterns, including 1) brain malformations or 1st and 2nd trimester patterns, which occur in utero 2) periventricular white matter changes, which are related to early 3rd trimester of pregnancy and preterm born infants, such as periventricular leukomalacia, defects following intraventricular or periventricular hemorrhage, and 3) cortical or deep grey matter lesions, which occur towards end of 3rd trimester or peri or neonatally, such as multicystic encephalomalacia and basal ganglia/thalamus lesions. Other patterns considered abnormal but not meeting above-mentioned criteria were classified as miscellaneous. Category of normal scans was also recorded. Classification of neurological abnormalities was done by a single senior radiologist. Investigations for inborn errors of metabolism and cytogenetics were advised if required on suspicion. We considered finding of periventricular leukomalacia, cystic encephalomalacia, and localized atrophy or gliosis with no other attributable cause or any of these with basal ganglia involvement as findings suggestive of birth asphyxia. Metabolic cause with definitive MRI patterns and malformations were not considered due to birth asphyxia. Statistical analysis was done using windows-based SPSS (statistical package for social sciences) version 17. Baseline demographic data was analyzed using students t-test (unpaired) for equality of means, and chi-square test or Fisher’s exact test for equality of proportions. Quantitative data was analyzed by sample t-test or ANOVA, and qualitative data by Fisher’s exact test or chi-square test.

## Results

Among the children studied, there was male preponderance (80.5%). Age at enrolment in the Child Development Clinic was 2-12 years. Most children were of term gestation (80.5%) and vaginal delivery (88.5%). Birth asphyxia was present in 41 (41.9%) cases. The most frequent clinical type of CP was spastic (n=91), followed by hypotonic (2), dystonic (2), and mixed types (3). Among spastic CP, most cases (n=68) had quadriplegic involvement, followed by hemiplegic (12), and diplegic (11). According to gross motor function classification of CP, 21 cases were in level I, 17 (level II), 23 (level III), and 37 (level IV). Epilepsy was associated abnormalities in 47 children (47.9%). The type of epilepsy were generalized tonicclonic (n=30), myoclonic (n=14), and complex partial seizure (n=3). Other abnormalities were high arched palate (24%), eye abnormalities (37%), hearing deficits (20%), and microcephaly (60.5%). Microcephaly did not vary significantly with birth asphyxia, sex, epilepsy, and gestation (p<0.05). Microcephaly was significantly more in cases of quadriplegic CP (p=0.003) as compared to other types of CP. Out of 98 children with magnetic resonance imaging (MRI), 94 children had abnormalities. Normal scans were seen in four children. The findings included periventricular white matter abnormalities (n=32, 34%), deep grey matter abnormalities (n=45, 47.8%), malformations (n=11, 11.7%), and miscellaneous lesions (n=6, 6.5%). Malformations included lissencephaly-3, focal cortical dysplasia-3, pachygyria-1, and craniovertrebral anomaly-1, arachnoid cyst-1, colpocephaly-2 (Table1). Types of neuroradiological abnormality did not vary significantly with birth asphyxia, gestation, microcephaly, and type of CP (p>0.05). Neuroimaging findings did not vary significantly with gestation (p=0.165) or type of CP (p=0.364). Neuroimaging findings of birth asphyxia, in comparison with rest did not vary significantly with birth asphyxia as documented at birth (p=0.678), sex (p=0.334), epilepsy (p=0.544) or microcephaly (p=0.527) (Table 2). Neuroimaging findings did not correlate with severity of CP.

## Discussion

The present study included neuroimaging findings of 98 children with CP. The high male to female ratio (4:1) is similar to other studies (1). The male preponderance may be attributed to reporting bias due to male preference and female neglect in local population. The presence of associated findings in our study was comparable to other Indian study (2). The incidences of various abnormalities were eye abnormalities: 37% vs. 35.8%, microcephaly: 60.5% vs. 46%, convulsions: 43.8% vs. 25.6% in our study versus those by Sharma et al., respectively (2). Incidence of hearing abnormalities in 20% of children in our study were comparable to 14% reported by Singhi et al. (1). Preponderance of spastic CP in our study is similar to other Indian and western studies (1,2,7). Spastic quadriplegic involvement is the most common type of CP in our study and other Indian reports as compared to western studies, where spastic diplegic is most common (1). Higher incidence of spastic diplegic CP may be attributed to higher preterm survival in developed countries compared to developing countries. There was no correlation between the types of CP with gestation as compared to western studies, which have shown diplegic CP is more common in preterm compared to term children. This may be due to lower incidence of preterm children (n=17) in our study. Higher incidence of convulsions in quadriparetic children, as in other studies, was not evident in our study (7-9). Role of perinatal complication especially birth asphyxia is controversial. Most western studies do not report birth asphyxia as cause of CP (10). However, our study as well as other Indian studies have reported presence of significant history and radiological evidence of asphyxia in these children (1,2). Neuroimaging abnormalities were seen in 95% of children, which is comparable to other western studies that have reported incidence of 86 to 91% (4,11,12). The neurological abnormalities in the present study were different compared to those of a systematic review by Krageloh-Mann et al. (4), which included 14 studies and 388 children. The incidence of various abnormalities in the present study vs. that of Krageloh-Mann et al. are malformations (11.4 % vs. 9%), periventricular changes (34% vs. 56%), and deep grey matter changes (47.8% vs. 18%), respectively. Presence of malformations in 11.4% of our cases was similar to other studies, which reported malformations in 9-42% (4,13). Malformations presenting as CP may have different management treatment, bearing on subsequent pregnancies and thus may require genetic counseling, and have medicolegal importance to obstetricians and neonatologists. Hence, the emphasis is on neuroimaging in children with clinically diagnosed CP. Periventricular changes and grey matter abnormalities were similar irrespective of gestation in our study as compared to western studies, which reported higher incidence of periventricular changes in preterm and grey matter abnormalities in children born at term gestation (4). However, due to lesser number of preterm (n=17) in our study incidence of periventricular changes was less. The weak correlation between radiological findings and clinical presentation of CP in our study compared to western studies, shows that further studies with greater sample size are required in our country. We presumed radiological findings, such as periventricular leukomalacia, cystic encephalomalacia, localized atrophy, and gliosis with or without basal ganglia involvement and with no other attributable cause, as findings suggestive of birth asphyxia. Metabolic cause with definitive MRI patterns, and malformations were not considered due to birth asphyxia. According to this, we had findings suggestive of birth asphyxia in 48.9% of cases. There was no correlation between presence of birth asphyxia and radiological evidence of birth asphyxia. Birth asphyxia was present in 41/98, but only 22 (54.8%) of them had evidence of birth asphyxia on neuroimaging. Similarly, 46 cases had evidence of birth asphyxia on imaging, of which only 22 (47.8%) had birth asphyxia as per definition. There is need to find out the cause of birth asphyxia by further investigation, since asphyxia may be the result of other cause e.g., cerebral malformations or inborn errors of metabolism at times not revealed by neuroimaging. The main limitation of our genetic and metabolic investigations was due to lack of facilities. These would have further enlightened us on the cause of birth asphyxia. As pathogenic events affecting the developing brain can cause abnormalities depending on the stage of brain development, MRI has high potential to elucidate type, extent, and possible time of brain damage in children with CP. Further studies are needed to confirm the same in Indian children. This further substantiates the recommendation of American Academy of Neurology to obtain neuroimaging findings on all children with CP, whenever feasible (14). 

**Table 1:** Neuroimaging Findings (n=98)

**MRI finding**	**Number (%)**
Normal	4 (4.4)
Periventricular white matter abnormalities/perventicular and intraventricular hemorrhage	32 (34)
Cortical or deep grey matter abnormalities including and basal ganglia changes and encephalomalacia	45 (47.8)
Brain malformations (lissencephaly-3, focal cortical dysplasia-4, pachygyria-1, arachnoid cyst-1, colpocephaly-2)	11 (11.7)
Miscellaneous (craniovertrebral anomaly-1, prominent sylvian fissures-2, gray white changes (leukodystrophy)-2, cerebellar atophy-1)	6 (6.4)

**Table 2:** Correlation of Clinical Parameters with Neuroimaging Features of Perinatal Asphyxia Versus Rest of the Abnormalities

**Clinical Parameters**		**Features of perinatal asphyxia n (%)**	**Other neuroimaging finding n (%)**	**p-** **value**
Birth asphyxia	Present	23 (47.6%)	21 (52.38%)	0.678
	absent	25 (46.15%)	29 (53.84%)	
Microcephaly	Present	27 (45.61%)	32 (54.38%)	0.527
	absent	21 (54.05%)	18 (45.95%)	
Gestation	Term	37 (45.56%)	44 (54.43%)	0.165
	Preterm	11 (66.67%)	6 (33.33%)	
Epilepsy	Present	22 (45.65%)	26 (54.34%)	0.544
	absent	26 (52.08%)	24 (47.91%)	

**Fig1:**
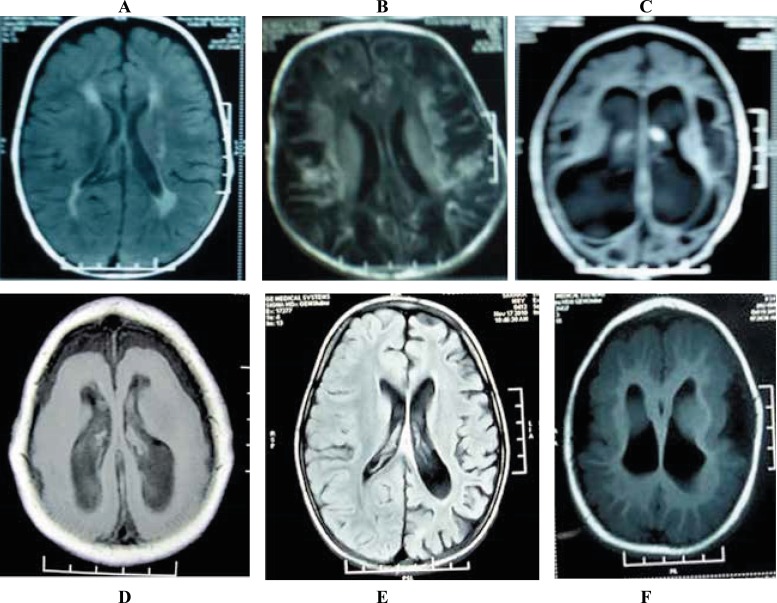
MRI findings of the studied children: A) Flair image axial section showing periventricular leukomalacia (hyperintensities around ventricles and peritrigonal areas), B) Flair image axial section showing cystic encephalomalacia (multiple cystic lesions and gliotic changes, and loss of grey and white matter). C) Flair image axial section showing porencephalic Cyst, D) T1-weighted image axial section showing lissencephaly (loss of sulci and gyri, smooth counter of both cerebral hemispheres), E) Hemiatrophy-flair image axial section showing cortical atrophy in left cerebral hemisphere and ex- vacuo dilatation of ventricles, F) T1-weighted image axial section showing pachygyria-thickening of cortical matter

## Contributors

AA conceptualized the study; all authors were involved in the collection of data, analysis and drafting of the manuscript; AA will act as guarantor for the study.

## Notes

**Funding: **None
